# Perception of the average size of multiple objects in chimpanzees (*Pan troglodytes*)

**DOI:** 10.1098/rspb.2017.0564

**Published:** 2017-08-23

**Authors:** Tomoko Imura, Fumito Kawakami, Nobu Shirai, Masaki Tomonaga

**Affiliations:** 1 Department of Information Systems, Faculty of Information Culture, Niigata University of International and Information Studies, Niigata 950-2292, Japan; 2 Wildlife Research Center of Kyoto University, Kyoto 606-8203, Japan; 3 Department of Psychology, Faculty of Humanities, Niigata University, Niigata 950-2181, Japan; 4 Primate Research Institute, Kyoto University, Inuyama, Aichi 484-8506, Japan

**Keywords:** perception, summary statistics, average, size, ensemble coding, chimpanzees

## Abstract

Humans can extract statistical information, such as the average size of a group of objects or the general emotion of faces in a crowd without paying attention to any individual object or face. To determine whether summary perception is unique to humans, we investigated the evolutional origins of this ability by assessing whether chimpanzees, which are closely related to humans, can also determine the average size of multiple visual objects. Five chimpanzees and 18 humans were able to choose the array in which the average size was larger, when presented with a pair of arrays, each containing 12 circles of different or the same sizes. Furthermore, both species were more accurate in judging the average size of arrays consisting of 12 circles of different or the same sizes than they were in judging the average size of arrays consisting of a single circle. Our findings could not be explained by the use of a strategy in which the chimpanzee detected the largest or smallest circle among those in the array. Our study provides the first evidence that chimpanzees can perceive the average size of multiple visual objects. This indicates that the ability to compute the statistical properties of a complex visual scene is not unique to humans, but is shared between both species.

## 1. Introduction

When humans see a group of apples in a supermarket, they are able to evaluate the whole display of multiple apples as well as any individual apple. Humans are remarkably good at computing statistical information such as average size [1–3], brightness [4], orientation [5,6], location [7], and number [8] of a group of objects, and average emotion [9], gender [9] and identity of faces in a crowd [10]. Even 4- to 5-year-old children can use average size to compare between two trees each with eight oranges of different sizes [11]. There is also evidence that humans can use other statistical information, such as variance [12], to represent a group of items. The ability to summarize large amounts of information presented in visual scenes is referred to as ensemble perception [3]. The use of statistics enables us to find regularity and predictability in complex visual scenes. It is not clear whether this ability is unique to humans.

Summary perception is an ecologically important ability for non-human primates, who need to extract essential information from whole visual scenes, particularly those living in large groups. For example, the animals often encounter groups of faces and share fruit from the trees with the group. Although they are not identical, groups of faces, trees and fruit have common features. If animals can integrate the common features of multiple objects into one category and calculate the average features, they can extract essential information from entire visual scenes efficiently. Although several vision science studies have demonstrated that humans can rapidly extract summary statistics from complex visual scenes, less is known about the ability of non-human animals to use statistical information. On this basis, we investigated ensemble perception in chimpanzees, which are closely related to humans, to explore the evolutional origins of using statistical information for processing global scenes.

Cumulative comparative studies have revealed several similarities in visual function between human and chimpanzee (e.g. contrast sensitivity [13], temporal characteristics of visual perception [14], early attentional processing [15–17] and working memory capacity [18]). Given the similarities in visual function between humans and chimpanzees, the mechanisms underlying ensemble perception in chimpanzees may be similar to that of humans. However, previous studies have found differences in human and non-human visual function, such as global information processing. For instance, the ability of humans to perceive a visual scene as a global configuration is superior to that of chimpanzees [19,20] and other primate species ([21,22], but see, [23]). Moreover, humans tend to perceive global configurations of hierarchically structured visual patterns (large letter made of small letters) before local elements [24], whereas chimpanzees show no preference in the hierarchical processing of compound stimuli [25]. These studies suggest that chimpanzees are more likely to attend to individual items in a complex visual scene than humans. Although it is unclear whether global processing and ensemble coding share common perceptual and/or neural mechanisms, they have common elements (perceiving configural patterns from multiple items and perceiving summary statistics from multiple items, respectively). Thus, there may be differences in the abilities of humans and chimpanzees to perform ensemble coding.

**Figure 1.**
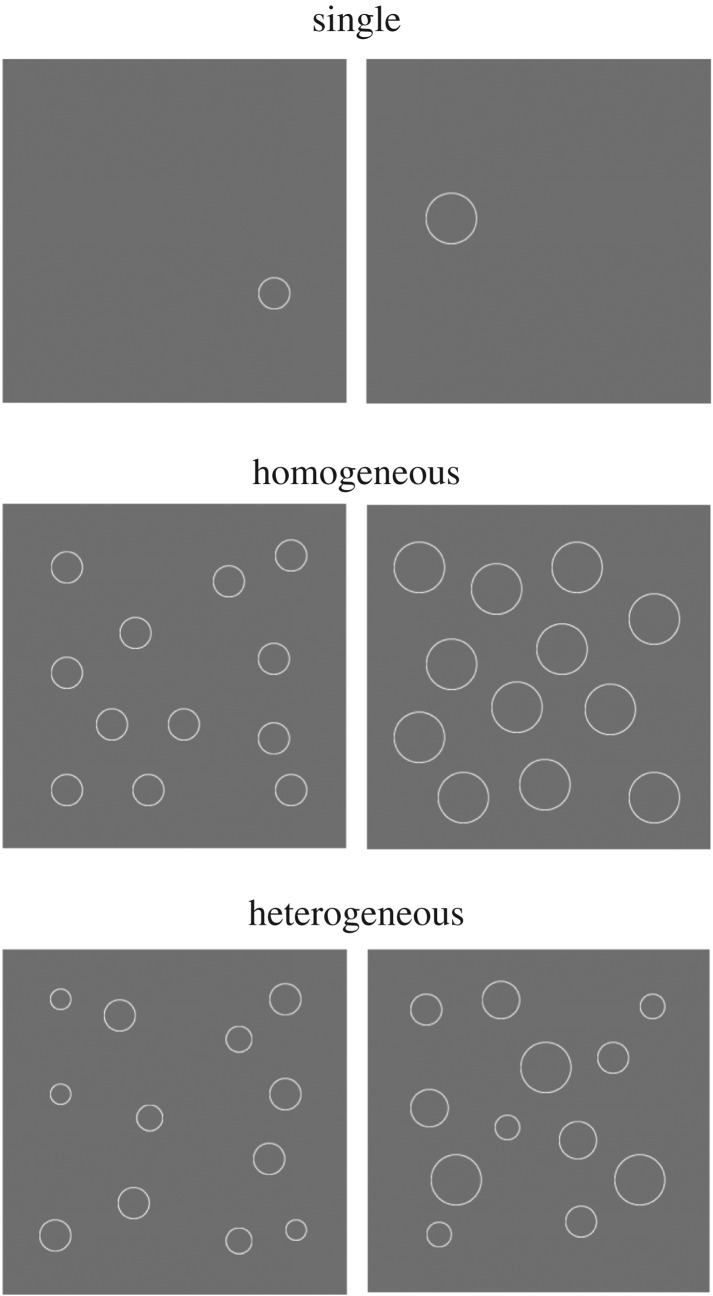
Examples of the stimuli used in Experiment 1. In the Single, Homogeneous and Heterogeneous conditions, the average diameter(s) of the circle(s) of one array (right side of the figure) was larger than that (those) of the other array. The figure shows examples of the stimuli in each condition, in which the size differences of circle(s) between the arrays were 20%.

Given these findings, we investigated whether ensemble coding ability in chimpanzees was similar to that in humans. To this end, we compared the ability to perceive the average size of objects in chimpanzees and humans because ensemble size perception is relatively well understood in human adults and children (e.g. [2,3,11]), and the ability to discriminate between object sizes has been investigated in chimpanzees [26]. In Experiment 1, chimpanzees and humans viewed a pair of arrays presented on a computer screen. Each array included one circle (Single condition) or 12 circles of the same (Homogeneous condition) or different sizes (Heterogeneous condition) against a grey background (figure 1). Chimpanzees and humans were required to touch the array having the larger average size. Intuitively, it should be easier to judge the size of individual circles in the two arrays under the Single condition than to judge the mean size of multiple circles in the two arrays under the Heterogeneous (Hetero) and Homogeneous (Homo) conditions. However, a previous study [3] using stimuli similar to those in our study found that the sensitivities for the mean size under Hetero and the Homo conditions were comparable with those under the Single condition. Therefore, if the participants in our study were able to judge the average size of multiple circles, performance under the Hetero and the Homo conditions would be expected to be equal, or at least not worse, than that under the Single condition. Conversely, if the participants were not able to judge the average size of multiple circles, performance under the Hetero and Homo conditions would be expected to be worse than that under the Single condition.

## 2. Experiment 1

### (a) Methods

#### (i) Participants

In total, five chimpanzees (*Pan troglodytes*; Ayumu, 14-year-old male, (ID Number of Great Ape Information Network: GAIN-ID, C-0608, https://shigen.nig.ac.jp/gain/ [27]); Chloe, 34-year-old female (GAIN-ID C-0441); Cleo, 14-year-old female (GAIN-ID C-0609); Pal, 14-year-old female (GAIN-ID C-0611) and Pendesa, 37-year-old female (GAIN-ID C-0095)) and 18 humans (14 males, mean age: 20.2 years and four females, mean age: 19.5 years) participated in Experiment 1. All chimpanzees lived in an enriched outdoor enclosure at the Primate Research Institute, Kyoto University, with other group members. They were fed fruits and vegetables three times daily during the experimental period. These chimpanzees had previously engaged in various computer-controlled perceptual and cognitive tasks (e.g. [13–21,26,28]).

The experimental protocol was approved by the Animal Welfare and Animal Care Committee of the Primate Research Institute, Kyoto University, and the chimpanzees were tested and cared for according to ‘The Guide for the Care and Use of Laboratory Primates, 3rd edition’ issued by the Ethics Committee of the Primate Research Institute, Kyoto University (2010). The human participants were undergraduate students, who participated in the experiment voluntarily. Informed consent was obtained from the human participants.

#### (ii) Apparatus

The experiments with chimpanzees were conducted in an experimental booth (1.8 × 2.15 × 1.75 m) adjacent to the chimpanzee facility. Stimuli were presented on an 18.1-inch colour LCD monitor with a touch-screen device (Iiyama, A4146D) located 40 cm above the floor. The resolution of the monitor was 1280 × 1024 pixels. The monitor was protected by a transparent Plexiglas panel, and participants could touch the monitor through an armhole (38.5 × 12 cm). The viewing distance was approximately 40 cm. A food dispenser (Biomedica BUF-310) delivered rewards to the chimpanzees following each correct trial via food trays attached below the monitor. All experimental events and responses were controlled by a computer (Hewlett-Packard Compaq, PM215AV) located outside the experimental booth.

#### (iii) Stimuli

We used three types of pairs of arrays to examine the ability to represent average size of multiple visual objects [3]. Each array consisted of one or 12 white circle(s) (123.00 cd cm^−2^) and a grey background (73.08 cd cm^−2^; figure 1). The circles in the array were positioned in a rectangular grid of four rows and four columns (3.81° × 3.81°). Each circle was centred in the cell with a random jitter between −0.5° and 0.5°. The position of the circles was different for every trial.

In the Single condition, the control condition, a single circle appeared in each array. This condition was included to confirm the ability to discriminate differences in size between two circles in chimpanzees and humans. In the Homo condition, 12 circles of the same size appeared in each array. In the Hetero condition, 12 circles of four different sizes appeared.

Under the Single and the Homo conditions, the diameter of the individual circle in one array, the standard stimulus, was fixed at 0.95°, 1.19°, 1.42° or 1.90°. The diameter of the individual circle of the other array, the comparison stimulus, ranged from 20% to 50% larger in increments of 10% (1.64°, 1.78°, 1.91° or 2.06°) in chimpanzees, and ranged from 5% to 20% larger with increments of 5% (1.44°, 1.51°, 1.58° or 1.64°) in humans. Under the Hetero condition, the array consisted of 12 circles including three circles of four different diameters. The variations in the circle sizes in each array were the same as those in a previous study [3]. The size of the grey background was 15.22° × 15.22°. The presentation duration of the circles was 1000 ms for chimpanzees, and 500 ms for humans.

For the humans, experimental parameters, including circle size and stimulus duration, were based on those used in previous studies [2,3]. However, we used different experimental parameters for the chimpanzees to minimize the effects of training on ensemble perception; thus, rather than training the chimpanzees in size discrimination to the level of that in humans, we used stimuli with larger size differences and longer stimulus durations (thus, decreasing the difficulty of discrimination).

#### (iv) Procedure

The trial was initiated after the participant touched the start key presented at the centre bottom of the monitor screen. A pair of arrays was presented, side by side. The task was to select the array having the circle(s) of larger average size.

Before the test sessions, the chimpanzees underwent three phases of training. First, they were presented with two arrays, each containing one circle, and were taught to touch the array with the larger circle (Single condition). In the second phase, the chimpanzees were shown two arrays containing multiple circles. All of the circles in each array were the same size; however, the circles in one array were larger than those in the other (Homo condition). In the third phase, the circles in each array were various sizes; however, the average size of the circles in one array was larger than that of the other array (Hetero condition).

In the first training phase (Single condition), the chimpanzees were trained to touch the array having the larger circle under the Single condition. During the training sessions, the chimpanzees were trained under trial conditions in which the difference in size of the circles included in the two arrays was 50%. Each session consisted of 24 trials. If the rate of the correct answers was greater than 75% per session, the difference between the sizes of the circles was reduced from 50% to 40%. The training session was continued until the rate of correct answers was greater than 75% in trials where the difference in the sizes of the circles was 30% and 20%. Then, they were trained under in trials involving all size differences. Each session consisted of 32 trials.

After training under the Single condition, they trained under the Homo and Hetero conditions. First, arrays consisting of six circles were used during the training sessions. As well as the Single condition, each training session consisted of 24 trials in which the differences in size of the circle included in the two arrays was 50%, 40%, 30% or 20%. Once their performance had reached the criteria, they were trained under trial conditions including all size differences. Each session consisted of 32 trials. The criteria were the same was those used under the Single condition. The total number of training sessions ranged from 12 to 24 (Ayumu: 13 sessions, Chloe: 24 sessions, Cleo: 14 sessions, Pal: 13 sessions, Pendesa: 12 sessions).

The test sessions included Single, Homo and Hetero conditions. Each test block consisted of 96 trials (4 sizes × 4 size differences × 2 correct positions × 3 conditions). Data from the first one of five blocks with chimpanzees and the one test block with humans were used for the analyses (results of the analysis for the full of five blocks in the chimpanzees are provided as electronic supplementary material, S1).

### (b) Results and discussion

**Figure 2.**
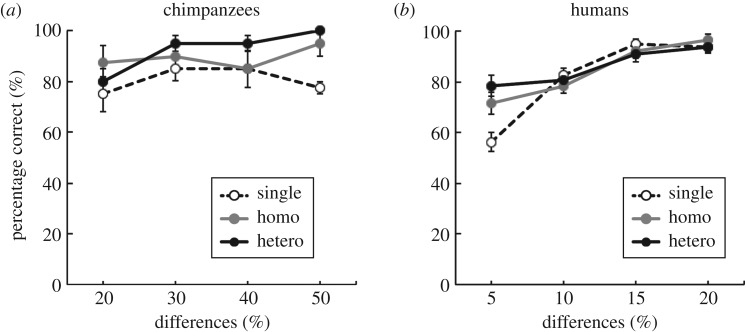
The proportion of choice of the array having the circles of larger average size in chimpanzees (*n* = 5) (*a*) and humans (*n* = 18) (*b*). The percentage of trials correctly completed under each condition as a function of differences in average sizes of circles between the arrays, averaged across participants. Standard errors are indicated by the error bars at each point.

The proportions of correct answers under the Single, Homo and Hetero conditions in Experiment 1 are shown in figure 2. The results showed that in chimpanzees and humans, performance under the Homo and Hetero conditions was higher than in the Single condition. Additionally, there was no difference in accuracy between the Homo and Hetero conditions. First, we analysed the proportion of correct answers for chimpanzees. A two-way ANOVA (conditions (3) × size differences (4)) revealed significant main effects of condition (*F*_2,8_ = 6.69, *p* < 0.05). The main effect of size (*F*_3,12_ = 2.53, *p* = 0.11) and the interaction between condition and size difference were not significant (*F*_6,24_ = 0.96, *p* = 0.47). Multiple comparison using Ryan's method revealed that there were significant differences between the Single condition and the other conditions (*p* < 0.05). This effect was sustained over five blocks and is therefore robust (see electronic supplementary material, data S1)

The same analyses were performed for the human results. A two-way ANOVA (conditions (3) × size differences (4)) revealed a significant main effect of size difference (*F*_3,51_ = 56.48, *p* < 0.001). The main effect of conditions was not significant (*F*_2,34_ = 2.35, *p* = 0.11). The interaction between condition and size difference was significant (*F*_6,102_ = 5.08, *p* < 0.001). The simple main effect of condition on accuracy at the smallest size differences was significant (*F*_2,136_ = 16.38, *p* < 0.001). Multiple comparison using Ryan's method revealed that there were significant differences between the Single condition and the other conditions (*p* < 0.05).

The findings of Experiment 1 suggest that chimpanzees may use average size when comparing the size of groups of circles included in an array. However, it remains unclear whether they used the average size or instead found an individual circle, such as the largest (or smallest) circle contained within the array under the Hetero condition. We examined this possibility in Experiment 2.

## 3. Experiment 2

### (a) Methods

#### (i) Participants

Four chimpanzees (except Ayumu) participated in Experiment 2.

#### (ii) Apparatus

The apparatus was the same as that used in Experiment 1.

#### (iii) Stimuli

**Figure 3.**
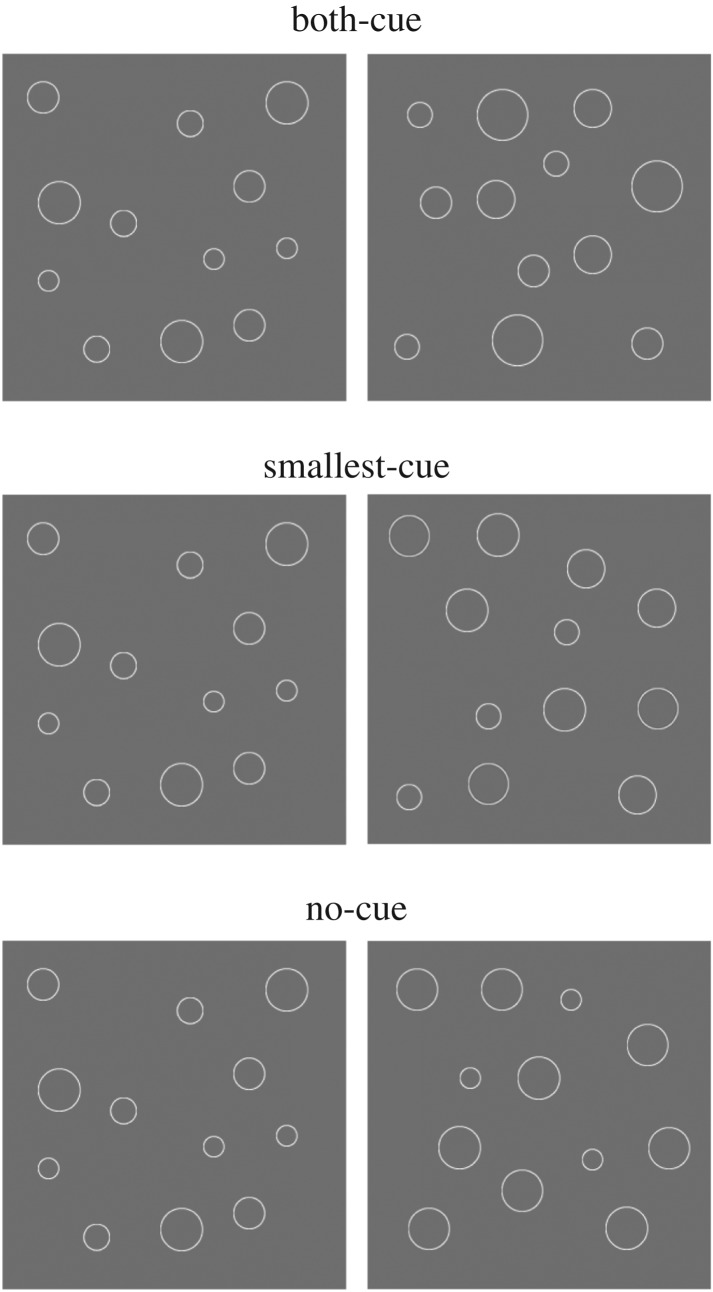
Examples of the stimuli used in Experiment 2. The test session consisted of Both-cue, Smallest-cue and No-cue conditions. The average diameters of 12 circles of one array (right side of the figure, comparison) were 20% larger than those of the other array.

We used three types of pairs of arrays to rule out the possibility that chimpanzees used the size of individual objects in Experiment 1. Each array consisted of 12 circles of four different sizes and a grey background (figure 3). The presentation duration of the circles was 1000 ms. We created three kinds of Hetero condition: the largest- and smallest-cues condition (i.e. the Both-cue condition), the Smallest-cue condition and the No-cue condition. In all three conditions, the average diameter of the 12 circles in the one array, the standard stimulus, was 1.37°, and those in the other array, the comparison stimulus, were 1.64°. Thus, the difference in the average size of the circles between the arrays was 20%. The diameter of the circles in the standard stimulus was fixed at 0.95°, 1.19°, 1.42° or 1.90°. The comparison stimulus was fixed at 1.14°, 1.43°, 1.70° or 2.28° in the Both-cue condition, at 1.14°, 1.71°, 1.80° or 1.90° in the Smallest-cue condition, and at 0.95°, 1.85°, 1.85° or 1.90° in the No-cue condition. While the chimpanzees could choose the array having the larger average size of circles by detecting the smallest circle in the Smallest condition, or the smallest and largest circle in the Both-cue condition, it could not choose the array accurately by using such a strategy in the No-cue condition. If chimpanzees detect the largest and/or smallest circle, the accuracy in the Both-cue condition would be expected to be superior to that in the Smallest-cue condition. The performance of the No-cue condition would be expected to be the worst of the three conditions. By contrast, if chimpanzees use average size to choose the array, there would be no difference in accuracy across the three conditions.

#### (iv) Procedure

The task procedure was identical to that of Experiment 1. The trial was initiated after the participant touched the start key presented at the centre bottom of the monitor screen. A pair of arrays was presented, side by side. The chimpanzees were required to select the array having the circle(s) of larger average size. In Experiment 2, the chimpanzees did not undertake training sessions because their performance had already met the criteria.

The test sessions included the Both-cue, Smallest-cue and No-cue conditions. Each test block consisted of 48 trials (2 correct positions × 3 conditions × 8 repetitions). Data from five test blocks were used in the analyses.

### (b) Results and discussion

**Figure 4.**
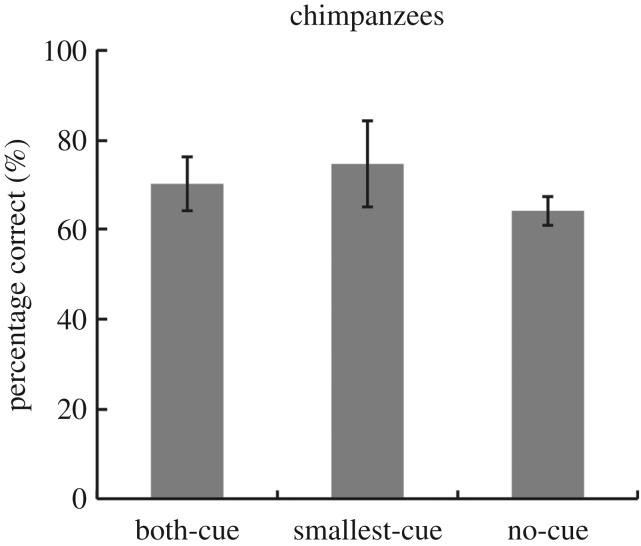
The proportion of choice of the array having circles of larger average size in chimpanzees (*n* = 4). The percentage of trials correctly completed under the three conditions, averaged across participants. Standard errors are indicated by the error bars on each column.

The proportions of correct answers in the Both-cue, Smallest-cue and No-cue conditions in Experiment 2 are shown in figure 4. One-way ANOVA (three conditions) confirmed that there was no difference in accuracy between the conditions (*F*_2,6_ = 1.51, *p* = 0.29). The results of Experiment 2 provide no evidence that chimpanzees were relying on the smallest circle or on both the smallest and the largest circles as cues for the experimental task.

Notably, the results were obtained from only four individuals, and there is a possibility that this analysis is underpowered. Although the statistical analyses revealed no significant differences in accuracy among the three experimental conditions, figure 4 suggests that performance under the No-cue condition was worse than that under the other two conditions. We examined the individual data and found that two of the four chimpanzees had lower correct answer rates under the No-cue condition than under the other two conditions (see the electronic supplementary material, data S2). Thus, it should be noted that it is possible that some chimpanzees might have used the size of individual objects as a basis for their responses.

Furthermore, it is possible that the chimpanzees may have used two alternative strategies of summary perception to discriminate between the arrays. First, it may be that differences in the variability of circle sizes between the arrays served as a cue for choice under the Smallest-cue condition. Thus, if variability were an effective cue for discrimination between the two arrays, performance would be best under the condition with the greatest variation between circle sizes in the standard and comparison stimuli. In Experiment 2, the variations in circle size in terms of standard deviations (s.d.) were 0.351 in the standard stimulus, 0.420 in the Both-cue array, 0.295 in the Smallest-cue array and 0.397 in the No-cue array. Furthermore, differences in the s.d. between the standard and comparison stimuli under the three conditions were 0.069 (standard versus Both-cue), 0.056 (standard versus Smallest-cue) and 0.046 (standard versus No-cue). Thus, the largest difference in circle size variation between the standard and comparison stimuli occurred under the Both-cue condition (see the electronic supplementary material, data S3). However, performance under the Both-cue condition was not significantly better than that under the other conditions, suggesting that differences in the variation of circle size between the two arrays might have little or no effect on the chimpanzees' performance under our experimental conditions.

Second, it is possible that under the No-cue condition, the chimpanzees counted the larger circles from a group of circles, because there was wide variability between circle sizes in each array. However, the results of Experiment 1 do not support this possibility: if the chimpanzees used this strategy, performance under the Single and the Homo conditions, in which the circle sizes did not vary, should have been better than that under the Hetero condition in Experiment 1. However, on the contrary, performance under the Hetero and Homo conditions was better than that under the Single condition. Therefore, there was no reason to infer that the chimpanzees used this strategy in Experiment 2.

Furthermore, to examine the possibility that chimpanzees compared the largest circle in each array under the Hetero condition, we re-analysed the Experiment 1 data. The combinations of the largest circles in the Hetero condition corresponded to those of the largest circle and the 20%, 30% 40% or 50% larger circles in the Single condition. We extracted these four types of trials from all Single condition trials and calculated the average correct answer rate (see the electronic supplementary material, data S4), and compared it with the correct answer rate under the Hetero condition. The analysis revealed that performance under the Hetero condition remained better than that under the Single condition (Hetero: 92.5%; Single: 70.0%), suggesting that the chimpanzees used ensemble perception rather than the largest circles in the array as a cue.

## 4. General discussion

These findings provide the first reported evidence that chimpanzees have the ability to extract summary statistics from global information. Our study suggests that the ability to compute the average size of multiple objects is shared by chimpanzees and humans.

Based on the findings of a previous study [3], we predicted that if chimpanzees were able to judge average size under the Hetero condition, their performance under the Hetero and Homo conditions would be equal to that under the Single condition. However, both humans and chimpanzees performed significantly better under the Hetero and Homo conditions than under the Single condition. The significantly higher accuracy for the Homo condition may be due to the redundant presentation of multiple identical circles [3]. Similarly, recent investigations of ensemble perception in humans found better performance in trials with multiple items [3,29]. In the chimpanzees, accuracy under the Homo and Hetero conditions was significantly higher than that under the Single condition regardless of circle size differences in each array, whereas in humans, the degree of accuracy was similar under all conditions except those with the smallest difference in circle sizes between arrays. Furthermore, performance in the humans improved more under the Single condition than under the Hetero and the Homo conditions as the size differences between arrays increased. Our findings are consistent with those of a previous study [3], suggesting that humans improve their visual sensitivity to perceive groups of objects by pooling across multiple representations and averaging out noise in visual representations [1,11].

In the current experiment, the chimpanzees underwent training sessions before the experimental tests. It is possible that the observed summary statistics reflect learning during the experimental protocol rather than the chimpanzees’ natural ability. In the future, we need to develop an experimental paradigm that addresses chimpanzees' abilities with regard to summary statistics but does so in the absence of training. Furthermore, in Experiment 2, although there was no statistically significant difference in the correct answer rate across the three conditions, the analysis involved data from only four participants. The individual data showed that two of the four chimpanzees had lower accuracy rates under the No-cue condition than under the other two conditions, implying that chimpanzees may have relied on other cues, such as variability. We need to examine this possibility in the future using more chimpanzees.

Finally, our findings suggest that both humans and chimpanzees show the ability to compute the average size of multiple objects; however, the mechanisms underlying ensemble perception of size remain unclear. In the future, determining whether chimpanzees can compute averages of other visual properties, such as brightness, orientation, location and number of groups of objects, as well as the average emotion, gender and identity of crowds will be important in understanding the mechanism underlying summary statistics.

## 5. Conclusion

This is the first reported study to demonstrate that non-human animals have the ability to perceive the average size of multiple visual objects. Both chimpanzees and humans could select which of two arrays, of heterogeneously and homogeneously sized circles, had the larger mean size more accurately than they could select which of two single circles was larger (Experiment 1). These findings are unlikely to be due to comparisons of individual circles within an array (Experiment 2). Our findings suggest that the size-averaging process, which is a means of statistically summarizing object features, is shared by non-human primate species.

## Data accessibility

All relevant data are available in the electronic supplementary material [30].
